# *Plasmodium vivax* VIR Proteins Are Targets of Naturally-Acquired Antibody and T Cell Immune Responses to Malaria in Pregnant Women

**DOI:** 10.1371/journal.pntd.0005009

**Published:** 2016-10-06

**Authors:** Pilar Requena, Edmilson Rui, Norma Padilla, Flor E. Martínez-Espinosa, Maria Eugenia Castellanos, Camila Bôtto-Menezes, Adriana Malheiro, Myriam Arévalo-Herrera, Swati Kochar, Sanjay K. Kochar, Dhanpat K. Kochar, Alexandra J. Umbers, Maria Ome-Kaius, Regina Wangnapi, Dhiraj Hans, Michela Menegon, Francesca Mateo, Sergi Sanz, Meghna Desai, Alfredo Mayor, Chetan C. Chitnis, Azucena Bardají, Ivo Mueller, Stephen Rogerson, Carlo Severini, Carmen Fernández-Becerra, Clara Menéndez, Hernando del Portillo, Carlota Dobaño

**Affiliations:** 1 ISGlobal, Barcelona Ctr. Int. Health Res. (CRESIB), Hospital Clínic - Universitat de Barcelona, Barcelona, Spain; 2 Department of Parasitology, Liverpool School of Tropical Medicine, Liverpool, United Kingdom; 3 Centro de Estudios en Salud, Universidad del Valle de Guatemala, Guatemala City, Guatemala; 4 Fundação de Medicina Tropical Dr. Heitor Vieira Dourado, Manaus, Amazonas, Brazil; 5 Instituto Leônidas e Maria Deane (ILMD/Fiocruz Amazonia), Brazil; 6 Universidade do Estado do Amazonas, Manaus, Amazonas, Brazil; 7 Instituto de Ciências Biológicas. Universidade Federal do Amazonas, Manaus, Brazil; 8 Caucaseco Scientific Research Center/Universidad del Valle, Cali, Colombia; 9 Department of Medicine, Medical College, Bikaner, Rajasthan, India; 10 Department of Medicine, University of Melbourne, Melbourne, Australia; 11 Papua New Guinea Institute of Medical Research, Madang, Papua New Guinea; 12 International Center for Genetic Engineering and Biotechnology, Delhi, India; 13 Department of Infectious, Parasitic and Immunomediated Diseases, Istituto Superiore di Sanità, Rome, Italy; 14 Centers for Disease Control and Prevention, Division of Parasitic Diseases and Malaria, Malaria Branch, Atlanta, Georgia, United States of America; 15 Walter and Eliza Hall Institute, Parkville, Australia; 16 ICREA, Barcelona, Spain; Queensland Institute of Medical Research, AUSTRALIA

## Abstract

*P*. *vivax* infection during pregnancy has been associated with poor outcomes such as anemia, low birth weight and congenital malaria, thus representing an important global health problem. However, no vaccine is currently available for its prevention. *Vir* genes were the first putative virulent factors associated with *P*. *vivax* infections, yet very few studies have examined their potential role as targets of immunity. We investigated the immunogenic properties of five VIR proteins and two long synthetic peptides containing conserved VIR sequences (PvLP1 and PvLP2) in the context of the PregVax cohort study including women from five malaria endemic countries: Brazil, Colombia, Guatemala, India and Papua New Guinea (PNG) at different timepoints during and after pregnancy. Antibody responses against all antigens were detected in all populations, with PNG women presenting the highest levels overall. *P*. *vivax* infection at sample collection time was positively associated with antibody levels against PvLP1 (fold-increase: 1.60 at recruitment -first antenatal visit-) and PvLP2 (fold-increase: 1.63 at delivery), and *P*. *falciparum* co-infection was found to increase those responses (for PvLP1 at recruitment, fold-increase: 2.25). Levels of IgG against two VIR proteins at delivery were associated with higher birth weight (27 g increase per duplicating antibody levels, p<0.05). Peripheral blood mononuclear cells from PNG uninfected pregnant women had significantly higher antigen-specific IFN-γ T_H_1 responses (p=0.006) and secreted less pro-inflammatory cytokines TNF and IL-6 after PvLP2 stimulation than *P*. *vivax*-infected women (p<0.05). These data demonstrate that VIR antigens induce the natural acquisition of antibody and T cell memory responses that might be important in immunity to *P*. *vivax* during pregnancy in very diverse geographical settings.

## Author Summary

Naturally-acquired antibody responses to novel recombinant proteins and synthetic peptides based on sequences from *P*. *vivax* VIR antigens were evaluated in women from five distinct geographical regions endemic for malaria, during and after pregnancy. Levels of IgG to VIR antigens were indicative of cumulative malaria exposure and increased with current *P*. *vivax* infection and *P*. *falciparum* co-infection. Antibody data were consistent with levels of malaria endemicity and current prevalence in the diverse geographical areas studied. In addition, the magnitude of IgG response to two VIR antigens at delivery was associated with higher birth weight. Furthermore, T cell responses to VIR antigens were naturally induced and their magnitude varied according to *P*. *vivax* infectious status. Peripheral blood mononuclear cells from uninfected pregnant women from a highly endemic area produced higher T_H_1 (IFN-γ) and lower pro-inflammatory cytokines (TNF and IL-6) upon stimulation with a long synthetic peptide representing conserved globular domains of VIR antigens than *P*. *vivax*-infected women. Data suggest that further investigation on these antigens as potential targets of immunity in naturally-exposed individuals is warranted.

## Introduction

Neglected for a long time, *P*. *vivax* malaria is raising more attention lately due to the increased recognition of its burden [1–4] and the renewed call for malaria elimination in endemic areas where *P*. *vivax* is an important source of malaria. Firstly, *P*. *vivax* is the most widely-spread of the human malaria parasites, with an at-risk population of 2.65 billion people [5]. Secondly, *P*. *vivax* infection is not as benign as traditionally believed, with severe malaria affecting a variety of population groups, including pregnant women in whom *P*. *vivax* infection has been associated with poor outcomes such as anemia, low birth weight (LBW) or congenital malaria [6–13].

The adverse consequences of malaria during pregnancy, the presence of parasites in the placenta and the molecular mechanisms of sequestration (parasite ligand and host receptor) have been well characterized in *P*. *falciparum* but to a lesser degree in the case of *P*. *vivax* infection. In *P*. *falciparum* infection during pregnancy, parasites may adhere to placental chondroitin sulphate A (CSA) through VAR2CSA, a member of the *P*. *falciparum* erythrocyte membrane protein 1 (*Pf*EMP-1) family [14,15]. Thus, susceptibility to placental malaria has largely been attributed to a set of *P*. *falciparum* strains expressing VAR2CSA. Host immunity to this particular parasite protein has been associated with exposure to or protection against *P*. *falciparum* infection during pregnancy [16,17]. There is controversy about *P*. *vivax* cytoadherence properties, although we have reported placental *P*. *vivax* monoinfections in Papua New Guinea (PNG) with no signs of placental inflammation [18]. Rosetting seems a frequent cytoadhesive phenotype during *P*. *vivax* infections, which may contribute to the development of anemia in pregnancy [19,20]. Nevertheless, a *P*. *vivax* orthologue of the *Pf*EMP-1 gene family and of VAR2CSA has not been described in parasites infecting pregnant women.

Like *P*. *falciparum*, the *P*. *vivax* genome contains subtelomeric multigene families. This includes the variant *vir* superfamily [21–23] with 295 *vir* pertaining to 10 subgroups [22,23]. From a structural point of view, *vir* genes differ greatly in size (156–2,316 bp in length) and number of exons (1–5). Unlike *Pf*EMP-1, VIR proteins represent an extremely diverse family clustered in subgroups, which suggests different subcellular localizations and functions. These functions may include immune evasion [22], although *P*. *vivax vir* genes do not undergo allelic exclusion in contrast to the clonal variant expression of *P*. *falciparum var* genes [24,25]. Moreover, VIR proteins can localize to the surface of infected reticulocytes [21,26] and induce the natural acquisition of antibodies after infection [24,27]. Nevertheless, the host immune responses to VIR proteins and their association with malaria outcomes have not yet been extensively characterized, even less in pregnancy, partly due to the extent of their diversity and the difficulty to express them as recombinant proteins for immunoassays.

We have partially overcome these two problems by using the wheat germ cell-free expression system and by producing two long synthetic peptides containing conserved VIR sequences (PvLP1 and PvLP2) based on the *P*. *vivax* line Sal-I. This strain is originally from El Salvador, which was monkey-adapted. To overcome the sequence polymorphisms, we determined conserved globular domains of presently unknown function to synthesize PvLP1 and PvLP2 for testing in immune-epidemiological field studies with parasites from different origins.

A recent meta-analysis has highlighted the necessity of cohort studies representing diverse geographical regions in the field of *P*. *vivax* infections, to increase the body of evidence for protective immunity [28]. As part of the PregVax project, a multicenter study aimed at describing the burden of *P*. *vivax* malaria in pregnancy, we set out to study naturally acquired immune responses to VIR proteins during pregnancy. Women from five different *P*. *vivax* endemic countries in America (Guatemala, Colombia, Brazil), Asia (India) and South Pacific (PNG) were enrolled and antigen-specific immune responses assessed. We used VIR-based recombinant proteins as well as PvLP1 and PvLP2 for antibody and cellular immunoassays. We demonstrate that despite the large diversity of *vir* sequences, women from all regions mounted antibody responses to the VIR antigens that increased with *P*. *vivax* infection and past exposure. Moreover, women from the highest endemic region (PNG) had detectable VIR-specific cellular memory immune responses with distinct patterns according with *P*. *vivax* infection status. Altogether, data indicate that VIR antigens might be targets of immunity to *P*. *vivax* during pregnancy.

## Results

### Expression of VIR proteins

**Fig 1:**
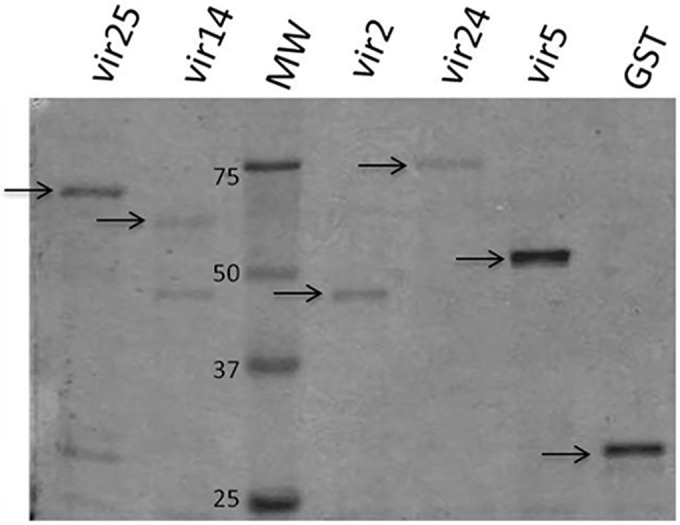
Expression of VIR proteins and GST in the wheat germ cell-free system. Five VIR proteins were expressed using the cell-free wheat germ system. Soluble extracts of each were analyzed by western blot using an anti-GST antibody. Purified soluble extracts were analyzed by SDS-PAGE and stained with Blue Coomassie Blue. Molecular weights (MW) markers in kilo-Daltons are shown in the third column, and soluble VIR-GST for the five fusion proteins of predicted sizes are marked with an arrow in columns 1,2,4,5 and 6. GST alone also expressed as a control, is shown in column 7.

A total of 16 *vir* genes were selected to be cloned and expressed. Twelve one-exon genes were selected for practical reasons as genomic DNA could be used as template (S1 Table). In addition, four *vir* genes were selected after a protein BLAST against VAR2CSA domains, presenting 18.8–30.6% protein identity (S2 Table). Because the *var2csa*-homology regions of *vir* genes were always located in exon 2, only this exon was cloned. Of these 16 *vir* genes, four one-exon *vir* genes were discarded for protein expression: two of them (PVX_006080 and PVX_241290) could not be cloned as the PCR reaction did not work and another two (PVX_045190 and PVX_106220) did not present the expected sequence after cloning. With the classical *E*. *coli* expression system, PVX_086890 and PVX_069690 were poorly induced; and PVX_015640, PVX_067190, PVX_090290 and PVX_115485 were insoluble. Attempts to purify the six remaining partially soluble VIR proteins expressed in *E*. *coli* resulted in very low yields and not completely clean proteins. Therefore, these six VIR proteins were cloned in the pIVEX vector and further expressed in the cell-free wheat germ system but PVX_112125 showed mutations in the sequence. Thus, five *vir* genes were successfully expressed in the wheat germ cell-free expression system: *vir25* (*vir25-related*, PVX_001610, group one-exon); *vir14* (*vir14-related*, PVX_101615, group one-exon); *vir2* (*vir2/15-like*, PVX_107750, group *var2csa* homology), *vir24* (*vir24-like*, PVX_093720, group var2csa homology) and *vir5* (*vir5-related*, PVX_124715, group *var2csa* homology). Soluble proteins were obtained of predicted sizes as detected by SDS-PAGE (Fig 1). Because expression of VIR proteins was not very productive, we designed two peptides containing conserved VIR sequences in order to perform the immunological assays.

### Characteristics of the study population

A total of 1,511 peripheral blood samples collected at different time points (recruitment, delivery or postpartum) corresponding to 1,056 women, were analyzed for antibody responses. Unfortunately many of our samples were not paired due to low follow-up rates. The study population characteristics at baseline by country are provided in S3 Table. The infection rates by country and time-point are provided in Table 1. The amount of VIR14, VIR2 and VIR24 proteins produced was not sufficient to measure antibodies to them in all samples, therefore enrolment-delivery-postpartum matching samples were prioritized. The numbers of plasma samples per country for which anti-VIR antibody data were generated against each antigen and at different time point are summarized in S4 Table. For cellular assays, 53 samples (any gestational age including delivery, 18 *P*. *vivax* PCR negative, 28 *P*. *vivax* PCR positive, 7 unknown infection status) from the PNG pregnant cohort were included in the analyses.

**Table 1:** Infection rate by timepoint and country in a random subset of samples for which microscopy and/or PCR data was available.

**RECRUITMENT**		**Brazil**	**Colombia**	**Guatemala**	**India**	**PNG**	**p-value**
*Pv* (microscopy)^a^	negative	117 (96%)	204 (96%)	172 (99%)	134 (100%)	135 (99%)	0.0120^c^
	positive	5 (4%)	8 (4%)	1 (1%)	0 (0%)	1 (1%)	
*Pv* (PCR)^a^	negative	112 (91%)	192 (90%)	148 (86%)	130 (99%)	71 (95%)	0.0008^b^
	positive	11 (9%)	21 (10%)	25 (14%)	1 (1%)	4 (5%)	
Any *Pv*	negative	121 (91%)	195 (90%)	148 (86%)	133 (99%)	133 (97%)	< 0.0001^b^
	positive	12 (9%)	22 (10%)	25 (14%)	1 (1%)	4 (3%)	
*Pf* (microscopy)^a^	negative	126 (100%)	202 (95%)	173 (100%)	134 (100%)	123 (90%)	< 0.0001^b^
	positive	0 (0%)	10 (5%)	0 (0%)	0 (0%)	13 (10%)	
*Pf* (PCR)^a^	negative	122 (99%)	195 (92%)	170 (98%)	-	69 (92%)	< 0.0001^c^
	positive	1 (1%)	18 (8%)	3 (2%)	-	6 (8%)	
Any *Pf*	negative	132 (99%)	199 (92%)	170 (98%)	134 (100%)	121 (88%)	< 0.0001^b^
	positive	1 (1%)	18 (8%)	3 (2%)	0 (0%)	16 (12%)	
**DELIVERY**		**Brazil**	**Colombia**	**Guatemala**	**India**	**PNG**	**p-value**
*Pv* (microscopy)^a^	negative	65 (93%)	105 (93%)	104 (99%)	100 (99%)	133 (99%)	0.0031^c^
	positive	5 (7%)	8 (7%)	1 (1%)	1 (1%)	1 (1%)	
*Pv* (PCR)^a^	negative	59 (88%)	83 (86%)	76 (82%)	88 (99%)	60 (92%)	0.0036^b^
	positive	8 (12%)	13 (14%)	17 (18%)	1 (1%)	5 (8%)	
Any *Pv*	negative	67 (89%)	102 (87%)	88 (84%)	99 (98%)	128 (96%)	0.0011^b^
	positive	8 (11%)	15 (13%)	17 (16%)	2 (2%)	6 (4%)	
*Pf* (microscopy)^a^	negative	72 (100%)	109 (96%)	105 (100%)	101 (100%)	128 (96%)	0.0075^c^
	positive	0 (0%)	5 (4%)	0 (0%)	0 (0%)	6 (4%)	
*Pf* (PCR)^a^	negative	67 (100%)	89 (93%)	90 (97%)	-	62 (95%)	< 0.0001^c^
	positive	0 (0%)	7 (7%)	3 (3%)	-	3 (5%)	
Any *Pf*	negative	75 (100%)	110 (94%)	102 (97%)	101 (100%)	126 (94%)	0.0103^c^
	positive	0 (0%)	7 (6%)	3 (3%)	0 (0%)	8 (6%)	
**POST-PARTUM**		**Brazil**	**Colombia**	**Guatemala**	**India**	**PNG**	**p-value**
*Pv* (microscopy)^a^	negative	38 (93%)	5 (62%)	59 (98%)	7 (58%)	53 (98%)	< 0.0001^b^
	positive	3 (7%)	3 (38%)	1 (2%)	5 (42%)	1 (2%)	
*Pv* (PCR)^a^	negative	38 (95%)	2 (40%)	37 (79%)	5 (38%)	29 (100%)	0.0001^b^
	positive	2 (5%)	3 (60%)	10 (21%)	8 (62%)	0 (0%)	
Any *Pv*	negative	39 (93%)	6 (60%)	50 (83%)	8 (62%)	54 (98%)	< 0.0001^c^
	positive	3 (7%)	4 (40%)	10 (17%)	5 (38%)	1 (2%)	
*Pf* (microscopy)^a^	negative	42 (100%)	10 (100%)	60 (100%)	13 (100%)	51 (94%)	0.1326^c^
	positive	0 (0%)	0 (0%)	0 (0%)	0 (0%)	3 (6%)	
*Pf* (PCR)^a^	negative	39 (98%)	4 (80%)	45 (96%)	-	28 (97%)	< 0.0001^b^
	positive	1 (2%)	1 (20%)	2 (4%)	-	1 (3%)	
Any *Pf*	negative	41 (98%)	9 (90%)	58 (97%)	13 (100%)	51 (93%)	0.5207 ^c^
	positive	1 (2%)	1 (10%)	2 (3%)	0 (0%)	4 (7%)	

^a^ number (percentage) in columns.

^b^ Chi-squared test.

^c^ Fisher’s exact test.

*Pv*: *P*. *vivax* infection. *Pf*: *P*. *falciparum* infection; PNG: Papua New Guinea.

### VIR antigens are targets of naturally-acquired antibodies

**Fig 2:**
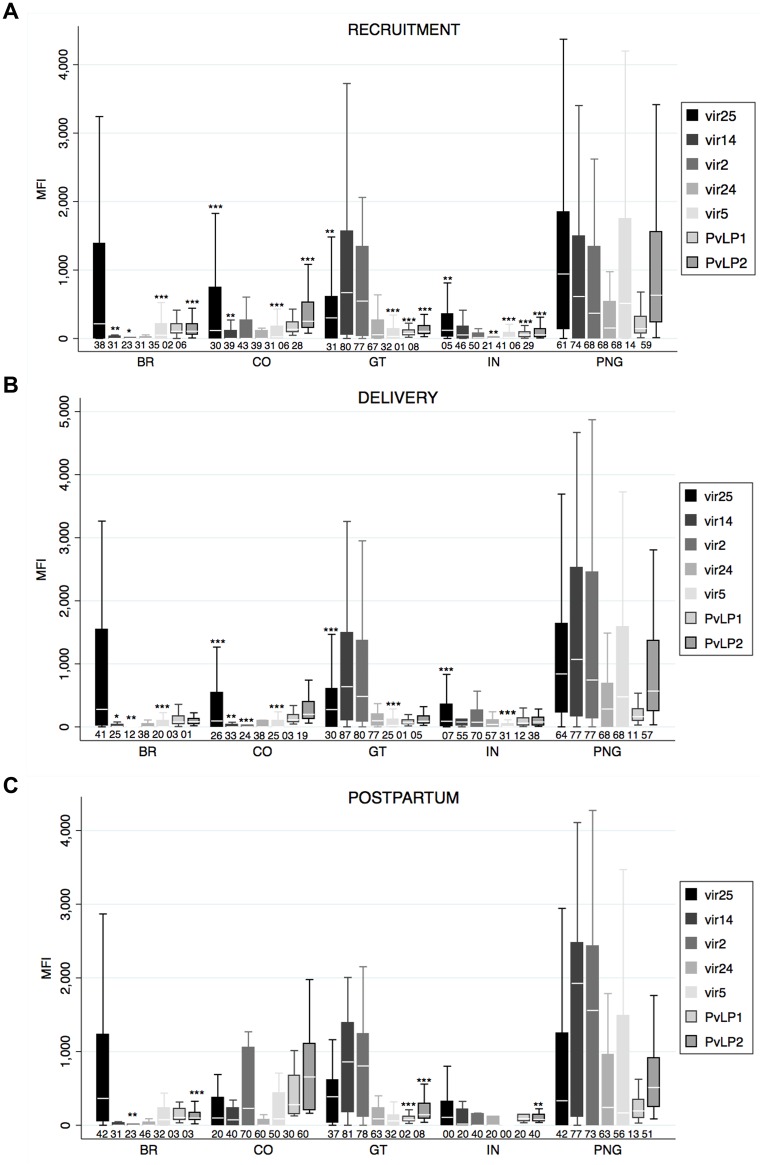
Antibody responses to VIR antigens. Human IgG antibodies against VIR proteins and peptides were detected by Luminex in peripheral blood at different timepoints: A) recruitment (first antenatal visit) (top panel), B) delivery (middle panel) and C) postpartum (after puerperium) (lower panel). Antibody levels are represented as median fluorescence intensity (MFI). Reactivity (MFI) against GST was subtracted from MFI values obtained against individual recombinant proteins. Median (white line), and 25^th^ and 75^th^ percentiles (lower and upper hinge respectively) are represented as boxes. Outside values are not displayed in the graph. Numbers below boxes represent percentage of positive responses, calculated as number of plasmas samples with MFI values above the mean plus 3 standard deviations of negative controls (cutoff). Cutoff for India samples was calculated with negative samples analyzed at this site and therefore differed from those used for the other sites. p corresponds to one-way ANOVA plus Bonferroni pairwise correction. Differences only displayed versus PNG. *p<0.05, **p<0.01, ***p<0.001 (adjusted p-values multiple comparisons). Sample size for each country, timepoint and antibody is provided in S4 Table. BR: Brazil; CO: Colombia; GT: Guatemala; IN: India; PNG: Papua New Guinea.

Antibody responses to all VIR antigens were detected (value above negative control cutoff) in all sites and timepoints, except VIR25 and VIR5 at postpartum in India (IN) (Fig 2). IgG levels to all VIR antigens differed among countries at all timepoints (except VIR25 at postpartum) (one-way ANOVA p<0.05). PNG presented the highest magnitudes and prevalence, followed by Guatemala (GT).

Overall, VIR25 appeared to be the most broadly recognized antigen, with significant responses across all endemicities, even in countries like Brazil (BR) and IN where IgG responses to the other VIR antigens were very low (Fig 2). In addition, VIR14 and VIR2 showed consistent and comparable responses in PNG and GT at all timepoints, stronger than those to VIR25, and for VIR2 a peak was also detected at postpartum in Colombia (CO). Finally, VIR5 and PvLP2 only appeared to be considerably recognized by plasma from PNG, and PvLP2 (and PvLP1) also in CO at postpartum. The lowest responses were measured for VIR24, only detected at moderate levels for PNG. Of note, anti-VIR seroprevalence was in range with other *P*. *vivax* antigens such as Pv200L: BR: 7%; CO: 35%; GT: 40%; IN: 5%; PNG: 76%.

At recruitment, IgG responses to VIR proteins were closely correlated (Table 2), but they correlated poorly with antibody responses to VIR synthetic peptides or other *P*. *vivax* antigens such as Pv200L, which corresponds to a fragment of the merozoite surface protein 1. PvLP2 presented the highest correlation with Pv200L (Table 2) and other *P*. *vivax* antigens.

**Table 2:** Correlation of IgG antibody responses to VIR antigens at recruitment.

VIR25	0.50						
VIR5	0.56	0.52					
VIR2	0.70	0.71	0.57				
VIR14	0.67	0.65	0.55	0.91			
PvLP1	0.08	0.15	0.17	-0.02	-0.08		
PvLP2	0.17	0.15	0.23	0.11	0.06	0.61	
Pv200L	0.24	0.12	0.22	0.25	0.21	0.47	0.62
	VIR24	VIR25	VIR5	VIR2	VIR14	PvLP1	PvLP2

Spearman’s correlation coefficient is displayed in the cells. The colour scale ranges between the dark grey (Spearman´s rho=|1|) and white (rho=0). Samples included in this analysis belonged to recruitment. Sample sizes for each antibody are provided in S4 Table.

We considered whether anti-VIR responses were due to cross reactivity with other *Plasmodium* antigens. To assess this, we studied the correlation between anti-VIR responses and antibody responses to 9 *P*. *vivax* and 6 *P*. *falciparum* additional antigens. Of note, low correlations were found between anti-VIR responses and other anti-*Plasmodium* responses, suggesting that there was no cross-reactivity (S5 table).

There were higher levels of anti-VIR24 IgGs at delivery, and more anti-VIR2, anti-VIR25 and anti-PvLP1 antibodies at postpartum, compared to recruitment levels, although overall differences using the Wald test were only significant for PvLP1 (S6 Table).

### Association between antibody levels and pregnancy variables and outcomes

We assessed how different pregnancy variables affected the IgG responses to VIR antigens. Antibody levels to VIR5 were significantly associated with gravidity (proportional differences by [category group of previous pregnancies] [0]: 1; [1–3]: 1.43, 95% CI: 0.91–2.25; [4+]: 0.71, 95% CI: 0.34–1.48, p=0.033). IgG responses to PvLP1 and PvLP2 were significantly associated with present malaria infections (Table 3). Of note, the association with co-infections (*P*. *vivax* and *P*. *falciparum*) was higher than with mono-infections (*P*. *vivax* alone). However, sample size for co-infections was small, especially at delivery, and these results should be considered cautiously. The magnitude of VIR-specific IgG response did not show associations with age and gestational age (p>0.05).

**Table 3:** Association of pregnancy variables with antibody levels.

		Recruitment^a^			Delivery^b^		
		Eff^c^	95% CI	p	Eff	95% CI	p
**PvLP1**	Pv-/Pf-	1		<0.001	1		0.033
	Pv+/Pf-	1.60	1.23; 2.07		1.11	0. 75; 1.66	
	Pv-/Pf+	1.20	0.87; 1.67		2.08	1.05; 4.11	
	Pv+/Pf+	2.25	1.01; 5.00		7.51	1.14; 49.44	
**PvLP2**	Pv-/Pf-	1		0.161	1		<0.001
	Pv+/Pf-	1.30	0.97; 1.73		1.63	1.09; 2.63	
	Pv-/Pf+	1.25	0.87; 1.80		4.20	2.04; 8.61	
	Pv+/Pf+	1.57	0.64; 3.83		11.27	1.54; 82.60	

Adjusted regression models were estimated for all combinations of positivity (+) and negativity (-) to *P*. *vivax* (Pv) and *P*. *falciparum* (Pf) infections.

^a^ Pv-/Pf- N=672; Pv+/Pf- N=52; Pv-/Pf+ N=33; Pv+/Pf+ N=5.

^b^ Pv-/Pf- N=425; Pv+/Pf- N=25; Pv-/Pf+ N=8; Pv+/Pf+ N=1.

^c^ Adjusted effect represents the fold-increase in antibody levels (MFI) per belonging to each category, after adjusting for the following variables: country of origin, age, gravidity (number of previous gestations), gestational age at recruitment and present malaria infection.

We also analyzed the association between antibody levels at recruitment and future infection (at delivery). Women with higher PvLP1 antibody levels at recruitment had a higher probability of having a *P*. *vivax* infection at delivery (per doubling antibody levels, OR=1.84, 95% CI=1.11; 3.04, p=0.017, adjusted analysis).

Finally, we studied the association between antibody levels and pregnancy outcomes, i.e. hemoglobin (Hb) levels and birth weight. A borderline significant positive association between PvLP2 antibody levels at recruitment and birth weight was observed by unadjusted regression analyses (Table 4). At delivery, IgG responses against two VIR proteins with homology to VAR2CSA (VIR2 and VIR24) were positively associated with birth weight in the adjusted analysis (Table 4). No associations were found between antibody levels and Hb levels at delivery (p>0.05).

**Table 4:** Association of antibody responses with birth weight.

	Recruitment						Delivery					
	Unadjusted			Adjusted			Unadjusted			Adjusted		
	Diff^a^	95%CI	p	Diff	95%CI	p	Diff	95%CI	p	Diff	95%CI	p
**VIR14**^b^	6.7	-16.71; 30.02	0.578	9.5	-13.85; 32.85	0.427	19.8	-5.96; 45.45	0.135	23.9	-1.91; 49.62	0.073
**VIR25**^c^	-5.0	-17.56; 7.48	0.431	-7.8	-20.70; 5.12	0.238	-10.5	-22.12; 1.15	0.078	-7.1	-19.17; 4.95	0.249
**VIR2**^b^	-1.4	-23.97; 21.09	0.900	6.8	-15.40; 29.05	0.549	17.6	-6.95; 42.18	0.163	**26.6**	**2.48; 50.72**	**0.033**
**VIR24**^b^	12.3	-12.65; 37.28	0.336	17.3	-8.05; 42.60	0.185	22.9	-2.29; 47.99	0.078	**27.0**	**1.93; 52.14**	**0.038**
**VIR5**^c^	8.6	-5.24; 22.43	0.224	4.6	-9.67; 18.90	0.527	5.0	-7.82; 17.78	0.446	4.8	-8.73; 18.32	0.488
**PvLP1**^c^	14.8	-23.79; 53.43	0.452	11.6	-28.11; 51.31	0.567	-16.9	-51.45; 17.58	0.337	-23.7	-59.48; 12.06	0.195
**PvLP2**^c^	**34.8**	**-1.67; 71.16**	**0.062**	24.7	-12.38; 61.68	0.193	0.6	-31.83; 33.07	0.970	4.4	-28.62; 37.37	0.795

Simple and adjusted regression models were estimated to establish the association between birth weight and the various IgG antibody levels at recruitment and at delivery, adjusting for the following variables: country of origin, age, gravidity (number of previous gestations), gestational age at recruitment and present malaria infection. Displayed in bold if p<0.07.

^a^ Difference in birth weight (g) per duplicating antibody levels.

^b^ N=104 at recruitment and N=101 at delivery.

^c^ N=382 at recruitment and N=449 at delivery.

### Cellular responses to PvLP1 and PvLP2

**Fig 3:**
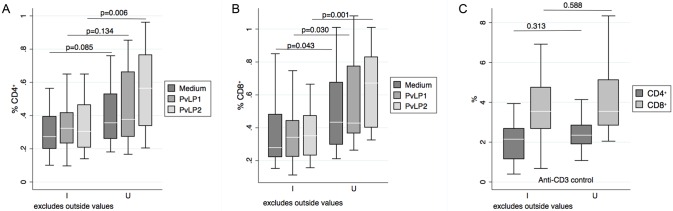
*In vitro* production of IFN-γ by peripheral blood mononuclear cells (PBMC) in response to VIR peptide stimulation. Intracellular production of IFN-γ by PBMCs was analyzed by flow cytometry. Box plots of the percentages of IFN-γ-producing cells from total CD4^+^ (A) or CD8^+^ (B) T cells are shown after PBMCs were cultured in the presence of PvLP1, PvLP2 or medium alone. In C), IFN-γ-producing cells from total CD4^+^ or CD8^+^ T cells are shown after culture in the presence of ant-CD3. IFN-γ production was compared between *P*. *vivax* infected (I, N=28) and uninfected (U, N=18) pregnant women; p corresponds to Mann-Whitney test. Median (middle line in white), and 25^th^ and 75^th^ percentiles (lower and upper hinge, respectively) are represented in the box. Outside values are excluded.

Peripheral blood mononuclear cells (PBMC) from PNG women with current *P*. *vivax* infection had a significantly lower percentage of IFN-γ-producing CD4^+^ and CD8^+^ T cells than uninfected women when stimulated with PvLP2, as assessed by intracellular cytokine staining by flow cytometry (Fig 3). No differences in % IFN-γ^+^ CD4^+^ T cells between infected and non-infected women were observed when stimulating PBMCs with PvLP1 or in the medium and anti-CD3 controls, and significant but much lower differences compared to PvLP2 stimulus were observed in % IFN-γ^+^ CD8^+^ T cells for PvLP1 and medium control (Fig 3).

We also measured the concentration of cytokines, chemokines and growth factors secreted in PBMC cultured either with medium, PvLP1 or PvLP2. Infected women produced more G-CSF and IL-4 than those from uninfected women, independently of the stimulus (Table 5). In addition, supernatants contained more IFN-γ when PBMC were cultured with medium or PvLP2, although the median value was the same, suggesting that differences were not very high. Of note, PBMCs stimulated only with PvLP2 secreted specifically more pro-inflammatory cytokines TNF, IL-6 and regulatory cytokine IL-10 in the infected group than in the uninfected group, although the difference did not reach statistical significance for IL-10 (p=0.062). No differences between the infected and uninfected cohorts were observed in the anti-CD3 control, although overall values in this positive control were higher than in the other three culture conditions.

**Table 5:** Median concentration (pg/mL) values for each cytokine, chemokine and growth factor in supernatants from infected (I) and uninfected (U) groups.

	MEDIUM			PvLP1			PvLP2			anti-CD3		
	U	I	p^a^	U	I	p	U	I	p	U	I	p
**EGF**	51	41	0.386	40	44	0.725	81	42	0.217	65	55	0.561
**Eotaxin**	13	3	0.248	13	3	0.333	13	3	0.349	13	3	0.228
**FGF**	18	17	0.259	15	17	0.282	15	16	0.541	36	30	0.379
**G-CSF**	**129**	**162**	**0.045**	**116**	**157**	**0.046**	**112**	**169**	**0.005**	211	221	0.903
**GM-CSF**	38	38	0.088	38	38	0.589	38	38	0.789	94	98	0.685
**HGF**	204	182	0.329	243	132	0.180	225	147	0.374	362	268	0.357
**IFN-α**	215	208	0.348	201	204	0.858	190	213	0.673	272	234	0.552
**IFN-γ**	**13**	**13**	**0.044**	13	13	0.088	**13**	**13**	**0.024**	868	607	0.291
**IL-10**	54	59	0.576	33	44	0.460	**35**	**71**	**0.062**	596	429	0.358
**IL-12**	56	55	0.891	56	42	0.916	55	52	0.983	168	112	0.626
**IL-13**	71	72	0.916	71	72	0.466	67	72	0.712	286	224	0.330
**IL-15**	147	86	0.142	313	156	0.093	313	147	0.113	366	329	0.358
**IL-17**	125	41	0.440	125	77	0.095	125	35	0.264	246	211	0.561
**IL-1β**	71	71	0.983	63	63	0.841	63	75	0.768	300	286	0.957
**IL-1Rα**	1503	1521	0.784	1446	1559	0.343	1503	1558	0.442	6192	4975	0.291
**IL-2**	**16**	**8**	**0.029**	9	8	0.217	11	7	0.164	1653	906	0.224
**IL-2R**	138	173	0.688	150	150	0.634	150	164	0.744	502	444	0.776
**IL-4**	**13**	**44**	**0.029**	**14**	**40**	**0.039**	**13**	**44**	**0.022**	229	154	0.379
**IL-5**	8	8	0.101	**8**	**8**	**0.014**	8	8	0.318	258	119	0.076
**IL-6**	1383	2182	0.225	817	1649	0.454	**1419**	**2406**	**0.046**	2643	2946	0.646
**IL-7**	75	75	0.946	75	75	0.465	75	75	0.674	76	102	0.348
**IL-8**	64000	64000	0.699	64000	64000	0.248	64000	64000	0.745	64000	64000	0.152
**IP10**	6	6	0.598	7	7	0.760	6	6	0.720	99	100	0.850
**MCP-1**	18640	13770	0.286	13911	14766	0.727	12752	17351	0.453	19201	18440	0.869
**MIG**	113	113	0.439	113	113	0.319	113	113	0.430	407	298	0.433
**MIP1-α**	1251	1597	0.924	866	1004	0.808	1291	1421	0.327	4893	5172	0.607
**MIP1-β**	1254	1152	0.792	678	1028	0.562	1101	1509	0.242	3855	5274	0.358
**Rantes**	203	158	0.620	104	121	0.907	80	140	0.393	454	387	0.570
**TNF**	62	47	0.792	32	39	0.422	**39**	**64**	**0.040**	2741	1568	0.111
**VEGF**	72	89	0.941	67	78	0.332	63	101	0.116	206	177	0.589

^a^ U-Mann-Whitney test. In bold if p<0.065.

G-CSF: granulocyte colony-stimulating factor, GM-CSF: granulocyte macrophage colony-stimulating factor, IFN: interferon, IL: interleukin, TNF: tumor necrosis factor, IP: IFN-γ-inducible protein, MCP: monocyte chemo attractant protein, MIG: monokine induced by IFN-γ, MIP: macrophage inflammatory protein, EGF: epidermal growth factor, FGF: fibroblast growth factor, HGF: hepatocyte growth factor, VEGF: vascular endothelial growth factor.

## Discussion

Considering the large genetic diversity of *P*. *vivax* strains [29] and the effect that polymorphisms in host genes such as HLA can have on immune responses to certain antigens [30], it is important to evaluate antibody and cellular immune responses to potential targets of immunity in different geographical populations. Here, significant levels of anti-VIR antibodies were detected in pregnant women from five countries with very diverse endemicity and transmission rates, further supporting the immunogenic properties of VIR antigens previously reported in non-pregnant Brazilian women [21,24,27]. This is remarkable if we consider that the Sal-I genome was used as a template for the production of all recombinant VIR proteins and suggests that despite the high sequence variability in the VIR proteins and the *P*. *vivax* circulating strains, B cell epitopes might be sufficiently conserved. This is further supported by the immunogenicity of long synthetic peptides representing conserved globular domains of VIR proteins, particularly PvLP2. We cannot, however, exclude the possibility that low responses to some VIR proteins in particular settings are due to lack of VIR expression or that they contain less B-cell epitopes as opposed to the inability to develop a VIR-specific immune response upon exposure to that variant.

Expressing recombinant *Plasmodium* proteins using different expression vectors has shown to be a challenging endeavor [31], especially achieving expression of soluble and correctly folded proteins is even more difficult. The cell-free wheat germ expression system used here has proven to be an excellent system to produce soluble and correctly folded proteins [32,33]. In fact, expression of enzymes from the human genome consistently showed that they retain enzyme activity [34]. In spite of these advantages, *vir* genes are highly AT-rich and several different attempts to express all the genes listed in S1 and S2 Tables using this or other systems such as cell-free and cell-based *E*. *coli* have failed.

PNG presented the highest intensity and prevalence of antibody responses against all antigens, despite *P*. *vivax* infection rates not being much higher at the time of the study within this cohort than in the other five countries [35]. The fact that asymptomatic infected women were not given treatment in PNG does not explain this difference, as the prevalence of *P*. *vivax* infection by microscopy in PNG was only 1%. Nevertheless, antibodies are a reflection of cumulative exposure, and PNG is indeed the country among the five with the highest malaria endemicity historically, even if during the PregVax study *P*. *vivax* prevalence was lower than in the past. In addition, regression analyses showed that co-infections with *P*. *falciparum* had a higher positive association with PvLP1 and PvLP2 antibody levels than *P*. *vivax* mono-infections. PNG had the highest *P*. *falciparum* microscopic infection rate in this cohort, suggesting these may boost anti-*P*. *vivax* responses. In our cohort we could rule out mostly although not totally undiagnosed submicroscopic co-infection and *vir* genes do not have orthologues in *P*. *falciparum*. It might be that this co-infection boosting effect is due to *P*. *vivax*-specific B cell bystander activation by noncognate T cells, which could be induced under conditions of persistent priming by *P*. *falciparum* antigens [36,37]. If this was the case, it may be interesting to consider this effect in programmatic terms regarding the search of a malaria vaccine. However, it is also possible that co-infections and higher antibody levels are just two parallel markers of higher previous exposure in some women.

Plasmas from GT also presented significant levels of IgG antibodies to various VIR proteins. This is consistent with *P*. *vivax* positivity rates by PCR at the population level, which were the highest in GT and PNG in the whole PregVax cohort. Infections in GT were largely submicroscopic but sufficient to induce detectable antibody responses. There was heterogeneity with regards to recognition of the VIR antigens among countries. VIR25 was the most broadly immunogenic, being recognized in distant geographical regions, suggesting the presence of conserved and/or cross-reactive epitopes within its sequences. However VIR14 and VIR2 induced the highest levels of antibodies, though restricted to the two most endemic countries. Those three proteins (VIR25, VIR14 and VIR2) appeared to induce longer-lived antibodies as they were clearly detected in populations with high infection rates in the past but low at the time of sampling. Antibodies to VIR24, VIR5 and PvLP2 were only clearly present in PNG, and this might indicate geographical diversity in immunogenicity of epitopes and/or their even longest-living nature. IgG antibodies against PvLP1 and PvLP2 were detected in all countries and timepoints but not at high levels. A peculiar pattern was observed in CO, where a significant increase in responses to most VIR antigens, but particularly VIR2 and PvLP2, occurred at postpartum. This likely reflects increased parasite prevalence at a population level at this time rather than a booster of VIR responses after pregnancy. However, at an individual level we did not find an association between antibodies to VIR proteins and infection status, which probably reflects the diversity of *vir* genes in the *P*. *vivax* genome [38]. In contrast, levels of PvLP1 and PvLP2 were associated with present vivax malaria infections. Both antibody levels but specially anti-PvLP2 correlated well with other markers of malaria exposure. This suggests that the design of these peptides (based on conserved sequences) might have helped overcome the problem of having a large and variable gene family. Thus, collectively the data showed that VIR antigens could be markers of exposure at a population level.

We also assessed association between antibodies and protection at the individual level, although this is often difficult as heterogeneity of exposure is not properly assessed and accounted for in field designs. In fact, higher PvLP1 antibodies at recruitment were associated with more risk of infection at delivery, being a correlate of risk rather than of immunity. We have previously reported that higher levels of antibodies in some individuals may indicate those who have had previous malaria episodes and are at higher risk of future episodes if past exposure is not well adjusted for [39,40]. Nevertheless, we also found some indications for a potential protective role of VIR antibodies in malaria in pregnancy outcomes: (i) a borderline significant positive association between PvLP2 antibody levels at recruitment and birth weight and (ii) a positive association of antibody levels to VIR2 and VIR24 (of partial sequence homology to *P*. *falciparum* VAR2CSA domains) at delivery and birth weight. Placental *P*. *vivax* infection has been reported [18], as well as *P*. *vivax* adhesion to CSA [19] and inhibition of *P*. *vivax* cytoadhesion using soluble CSA [41]. However, whether VIR2 and VIR24 proteins also bind CSA and are implicated in vivax malaria during pregnancy remains speculative. Unfortunately, our study was not designed to demonstrate any protective role of antibody responses to VIR antigens in vivax malaria and therefore we cannot draw any conclusion.

We present some evidence supporting a relationship between antigen-specific cytokine responses, infection and immunity in PNG pregnant women. Our data show lower PvLP2-specific IFN-γ^+^ CD4^+^ T cell frequencies and higher secretion of TNF, IL-6 and IL-10, in *P*. *vivax*-infected pregnant women compared to uninfected women and this was not seen for PvLP1 or the control stimuli. IFN-γ has been shown to be essential for controlling experimental malaria infections in mice (reviewed in [42]) and clinical *P*. *falciparum* infections in humans [43,44], and IL-10 is a key regulatory cytokine that prevents excessive inflammation but might contribute to the lack of control of infections. Thus, the fact that non-infected women had higher PvLP2-specific T_H_1 cell frequencies and lower IL-10 production could mean that cellular responses induced by this antigen (for instance by a potential vaccine) could help in controlling *vivax* infections. Nevertheless, we also observed PvLP2-specific increase of pro-inflammatory cytokines IL-6 and TNF in infected women. IL-6 has been shown to skew T cell differentiation towards T_H_2 and T_H_17 [45], which would explain why we observe a decrease of T_H_1 frequencies. Thus we can assume that VIR epitopes present in PvLP2 trigger the natural acquisition of cellular memory immune responses, but whether these are protective or just markers of exposure can not be concluded from the data presented.

This study presents some limitations. First, samples were not fully paired and sample size was different for some antigens/analyses. Unfortunately, many of these women lived in rural areas far from the hospital. It is highly complex to get full attendance to all antenatal clinics and, after puerperium, is even more complicated. In spite of this, we believe the cohort is quite unique and very valuable to demonstrate the immunogenicity of VIR antigens in different geographical settings. Second, due to its exploratory nature, we did not have statistical power to demonstrate strong associations between anti-VIR immune responses and protection against infection nor poor outcomes, as it was designed to be a first descriptive investigation of adaptive immune response (antibody and T cells) to VIR antigens during pregnancy. Third, multiple comparisons were not corrected for in all statistical assays and results are interpreted for internal coherence and biological plausibility.

In summary, we present the first comprehensive study on immune responses to VIR antigens demonstrating that VIR sequences are the target of the natural acquisition of antibody and cellular responses affected by exposure to malaria infection in five distinct endemic areas. VIR 25 seems to be broadly recognized and we demonstrate that PvLP1 and PvLP2 can be used to profile antibody and cellular immune responses to VIR sequences, overcoming the problem of the large number of diverse VIR proteins. Based on our findings and the large burden of *vivax* malaria, we believe that larger prospective cohort immune epidemiological studies are needed to specifically address whether VIR-based antigens are targets of protective immunity against the neglected *P*. *vivax* parasite and could be considered as candidates for vaccine development towards malaria elimination.

## Methods

### Study design and population

This study was performed in the context of the PregVax project (FP7-HEALTH-201588, www.pregvax.net), a health facility-based cohort study of pregnant women to describe the burden and impact of *P*. *vivax* in pregnancy, conducted between 2008 and 2012 in five endemic countries: BR, CO, GT, IN and PNG. Approximately 2,000 women per country were enrolled at the first antenatal visit (recruitment), and followed up until delivery. Symptomatic *Plasmodium spp*. infections at any time during pregnancy were also recorded though passive case detection. A random subpopulation corresponding to 10% of the entire PregVax cohort was allocated to the “immunology cohort” and was further followed up until at least 10 weeks after delivery (postpartum group). In all visits, Hb levels, *P*. *vivax* and *P*. *falciparum* parasitemias by blood smear and malaria symptoms were assessed. Giemsa-stained thick and thin blood slides were read onsite following WHO standard quality-controlled procedures to establish parasite presence. External validation of a subsample of blood slides from each country was done at the Hospital Clinic and at the Hospital Sant Joan de Deu, in Barcelona, Spain. Birth weight was recorded. Women with a positive smear were treated according to national guidelines, except in PNG where blood smears could not be read at the moment of the visit for logistical reasons (only symptomatic women were thus treated after confirmation of infection by rapid diagnostic test). The protocol was approved by the national and/or local ethics committees of each site, the CDC IRB (USA) and the Hospital Clinic Ethics Review Committee (Barcelona, Spain). Written informed consent was obtained from all study participants.

A venous blood sample (5–10 mL) was collected aseptically in heparinized tubes from the “immunology cohort” at recruitment, delivery and postpartum visits. Submicroscopic *P*. *vivax* and *P*. *falciparum* infections were also determined by real time-polymerase chain reaction (PCR), except for the Indian samples, where only *P*. *vivax* infection was examined. Submicroscopic infections were only analyzed in a random sub-sample of the cohort. Additionally, blood samples (10 mL) were collected from 39 malaria naïve donors at the blood bank in Hospital Clinic (Barcelona, Spain), and used as negative controls.

### Processing of plasma and PBMC

Plasma was separated by centrifugation and stored at -80°C. Blood cells from PNG were further fractioned in a density gradient medium (Histopaque-1077, Sigma-Aldrich) to obtain PBMCs and stored in liquid nitrogen. Samples from GT, CO, BR and PNG were analyzed at ISGlobal (Barcelona, Spain) while plasmas from IN were analyzed in Delhi.

### Recombinant proteins and long synthetic peptides

*Vir* genes were amplified from genomic DNA (Sal I strain) by PCR using “PCR Supermix” (Life Technologies). PCR products were introduced in the pIVEX1.4d vector (Roche) previously modified by inserting glutathione S-transferase (GST) after the 6xHis tag sequence. Authenticity of all clones encoding GST-VIR fusion proteins was confirmed by double-strand sequencing before expression in the wheat germ system. Thus, GST-fusion proteins contain open reading frames encoding the predicted VIR proteins.

Primers used for gene amplification are listed in S1 and S2 Tables.

Proteins were expressed with a GST tag using the wheat germ cell-free system as described [46]. Expressed proteins were purified on GST SpinTrap purification columns (GE Healthcare), and eluted proteins were dialyzed in phosphate buffered saline (Tube-O-DIALYZER, GBiosciences). GST was also expressed separately for immune-reactivity control. Pv200L (*P*. *vivax* merozoite surface protein 1, fragment 121–416) was produced as previously described [47]. The rest of *Plasmodium* antigens were produced as described previously [48]. The design and synthesis of *P*. *vivax* long synthetic peptides (PvLP) representing conserved central core (PvLP1) and C-terminal (PvLP2) VIR motifs, has been reported previously [26]. The sequences are detailed in S7 Table.

### Quantification of IgG antibodies

Measurement of plasma IgG antibodies was performed by multiplex suspension array using the Luminex technology, as described [46]. MagPlex magnetic carboxylated microspheres (Luminex Corporation, TX, USA) were covalently coated with 3 μg of protein/peptide per 1.1–1.4 million beads following manufacturer’s instructions. Beads were quantified in a Guava Flow Cytometer (Millipore) and mixed in equal amounts. A unique batch of microspheres was prepared for the whole study, including the samples analyzed in IN. Circa 1000 beads per analyte were incubated with plasma (1:100 dilution) in duplicates, and subsequently with anti-human IgG-biotin (Sigma-Aldrich), followed by streptavidin-conjugated R-PE (Fluka, Madrid, Spain). Beads were acquired on the BioPlex100 system (Bio-Rad, Hercules, CA), and results expressed as median fluorescence intensity of duplicates. Value against GST alone was subtracted for VIR proteins. Raw GST values are presented in S1 Fig. Cross-reactivity was ruled out in a pilot study analyzing a subset of plasmas in singleplex and multiplex. Samples in IN were analyzed with identical protocols and instruments.

### Cellular stimulation assays

Except where indicated, all reagents were purchased from BD Biosciences. PBMCs were thawed, rested for 10–12 h and viability assessed with Guava ViaCount Reagent (Millipore). Only samples with viability >70% were used for assays. Half a million cells per well were resuspended in *RPMI-1640* medium plus 10% fetal bovine serum (culture medium) and incubated with PvLP1 or PvLP2 (5 μg/mL). Culture medium was used as negative control and anti-CD3 as the positive control. After 12 h, an aliquot of 30 μL of culture medium supernatant was collected to measure secreted cytokines, while an equal volume of media containing GolgiPlug was added for additional (4 h) incubation. PBMCs were stained with LIVE/DEAD Fixable Violet Dead (Life Technologies), anti-CD14 Pacific Blue, anti-CD19 Horizon V450, anti-CD4 allophycocyanin (APC) and anti-CD8 Peridinin Chlorophyll Protein Complex (PerCP). After washing, cells were fixed and permeabilized with Cytofix/Cytoperm, and incubated with anti-CD3 phycoerythrin (PE)-Cy7, anti-interferon (IFN)-γ PE and anti-CD69 fluorescein isothiocyanate (FITC). Cells were acquired in a LSRFortessa flow cytometer and data were analyzed by FlowJo (FlowJo LLC, OR, USA). Gating strategy is provided in S2 Fig. Supernatants were frozen at -80°C until Luminex analysis with the Cytokine Magnetic 30-Plex Panel (Invitrogen), according to manufacturer’s instructions.

### *Plasmodium spp*. detection by PCR

Samples from BR, CO, GT, and half of the samples from PNG were analyzed at the Istituto Superiore di Sanità (Rome, Italy), as described [12]. The threshold for positivity for each species was established as a cycle threshold<45, according to negative controls. *P*. *vivax* diagnosis for IN samples was performed in Delhi following Rome’s protocol adapted for the instrument sensitivity (3^rd^ step amplification 72°C for 25 sec instead of 72°C for 5 sec). Approximately half of the PNG samples were analyzed for submicroscopic infections in Madang, following a similar protocol to Rome’s [49], except that the threshold for positivity for each species was established as cycle threshold<40, according to negative controls. DNA was extracted from whole blood-spot filter paper.

### Definitions and statistical methods

Any *Plasmodium* infection was defined as a positive smear by microscopy and/or positive PCR. One-way ANOVA test was used to evaluate the differences in antibody levels among countries, and Chi-squared or Fisher’s exact tests to evaluate the differences in percentages of individuals with a positive antibody response (values above the mean plus 3 standard deviations [SD] of Spanish controls, cutoff). To assess the differences on antibody levels between non-pregnant and pregnant women at recruitment and delivery, multilevel mixed-effects linear regressions were estimated with the samples from the five countries. Timepoint (recruitment, delivery and postpartum) was the fixed independent variable, while inter-site (country of origin) and inter-subject variability were estimated as random parts. To study the association between antibody levels and pregnancy variables, univariate (only adjusted for country of origin) and multivariate linear regression models were estimated with the variables country, age, gestational age, gravidity (number of previous gestations) and *P*. *vivax* or *P*. *falciparum* infection during pregnancy (only accounted past or present infections but not future infections). The correlation between IgG responses to different antigens was evaluated with the Spearman's rank test. The association between IgG levels at enrolment and future malaria infections was evaluated with logistic regression models. The association between antibody responses at enrolment and delivery, and Hb levels at delivery and birth weight, were analyzed using univariate and multivariate linear regression models, adjusted by country, Hb at recruitment, gestational age at recruitment, age, gravidity and past or present *Plasmodium* infection during pregnancy. For the cellular and cytokine analyses, deviation from normality was tested using the Skewness and kurtosis test. Because none of the variables except IL-13 presented a normal distribution, data was presented as medians and comparisons between groups were done using the U-Mann-Whitney test. Cytokine/chemokine production in culture supernatants of unstimulated samples (medium) was not subtracted from the stimulated samples but shown side by side as it is possibly biologically relevant. Significance was defined at p<0.05. Crude p values are interpreted for internal coherence, consistency of results and biological plausibility. Analyses were performed using Stata/SE 10.1 (College Station, TX, USA).

## Supporting Information

S1 Fig Human IgG antibodies against GST were detected by Luminex in peripheral plasma at recruitment. Antibody levels are represented as median fluorescence intensity (MFI). Median (white line), and 25^th^ and 75^th^ percentiles (lower and upper hinge respectively) are represented in the box. Outside values are not displayed in the graph.
(GIF)

S2 Fig Gating strategy. After exclusion of debris and doublets, lymphocytes were displayed according to CD3 expression and a dump channel containing a viability marker, CD14 and CD19. Live CD3^+^ T cells were then gated for CD4^+^ and CD8^+^ and intracytoplasmic expression of IFN-γ and CD69 was assessed in each of the populations.
(JPG)

S1 Table Characteristics of the one-exon vir genes and proteins selected for cloning and expression, and primer sequences used for gDNA amplification. (DOCX)

S2 Table Characteristics of the var2csa-like vir genes and proteins selected for cloning and expression, and primer sequences used for gDNA amplification. (DOCX)

S3 Table Baseline characteristics of study population. (DOCX)

S4 Table Number of samples for which anti-VIR antibody responses were analyzed by country, timepoint and antigen. (DOCX)

S5 Table Correlation of anti-VIR responses with other anti-*Plasmodium* responses. (DOCX)

S6 Table Effect of time of bleeding on antibody levels. (DOCX)

S7 Table Peptide sequences of the long peptides (PvLP). P. vivax long synthetic peptides (PvLP) representing conserved central core (PvLP1) and C-terminal (PvLP2) VIR motifs. Derivatized diethylene glycol (DEG,Merck Chemicals,Nottingham, UK) was inserted in between the different individual segments.
(DOCX)

S1 Checklist STROBE checklist for cohort studies. (DOC)
