# Supplementary figures and images for: Plasmodium vivax VIR Proteins Are Targets of Naturally-Acquired Antibody and T Cell Immune Responses to Malaria in Pregnant Women
Source: PLoS Negl Trop Dis. 2016 Oct 6;10(10):e0005009. doi: 10.1371/journal.pntd.0005009 (PMC5053494; doi:10.1371/journal.pntd.0005009)

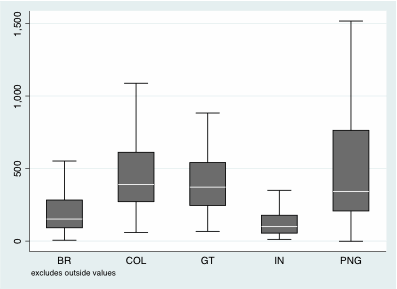

Supplement: S1 Fig — Antibody levels are represented as median fluorescence intensity (MFI). Median (white line), and 25th and 75th percentiles (lower and upper hinge respectively) are represented in the box. Outside values are not displayed in the graph. (GIF) [file pntd.0005009.s001.gif]

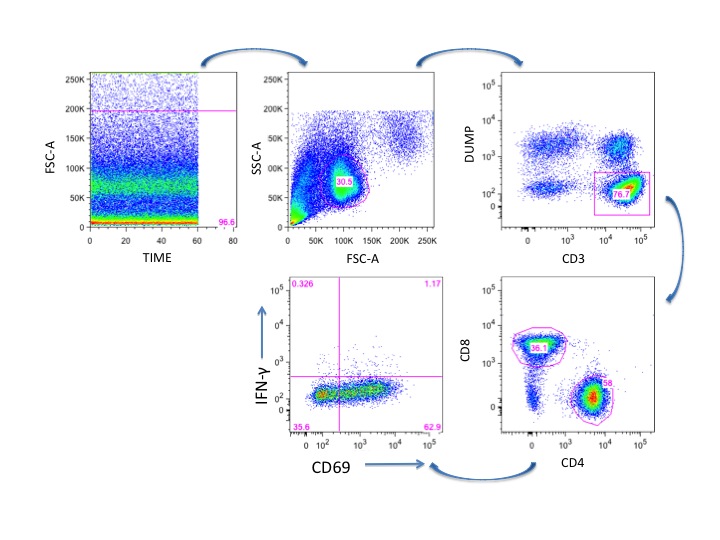

Supplement: S2 Fig — After exclusion of debris and doublets, lymphocytes were displayed according to CD3 expression and a dump channel containing a viability marker, CD14 and CD19. Live CD3+ T cells were then gated for CD4+ and CD8+ and intracytoplasmic expression of IFN-γ and CD69 was assessed in each of the populations. (JPG) [file pntd.0005009.s002.jpg]
