# Supplementary material for: Plasmodium vivax VIR Proteins Are Targets of Naturally-Acquired Antibody and T Cell Immune Responses to Malaria in Pregnant Women
Source: PLoS Negl Trop Dis. 2016 Oct 6;10(10):e0005009. doi: 10.1371/journal.pntd.0005009 (PMC5053494; doi:10.1371/journal.pntd.0005009)
Supplement: S1 Table — (DOCX) [file pntd.0005009.s003.docx]

**S1 Table**. **Characteristics of the one-exon *vir* genes and proteins selected for cloning and expression, and primer sequences used for gDNA amplification**.

| **name** | **id** | **exons** | **aa** | **MW** | **TM** | **pI** | **primer sequence** | |
| --- | --- | --- | --- | --- | --- | --- | --- | --- |
| **VIR25-reated** | **PVX_001610** | 1 | 338 | 38699 | 1 | 5.73 | f | cacccgcggccgcATGcagtatgaaaaaaattgtcctc |
|  |  |  |  |  |  |  | r | gcgctcgagTTActttaccttgtaaaaaatgaaga |
| **VIR18-related** | **PVX_006080** | 1 | 577 | 63860 | 0 | 8.49 | f | cacccgcggccgcATGacgaattggcttactggaagat |
|  |  |  |  |  |  |  | r | gcgctcgagTTAacaactgtgtatgcaattggtattat |
| **VIR18-related** | **PVX_015640** | 1 | 427 | 47068 | 0 | 7.28 | f | cacccgcggccgcATGtcgaggtggactggaacatctagg |
|  |  |  |  |  |  |  | r | gcgctcgagTTAtaactcatacgtcattttacaatta |
| **VIR18-related** | **PVX_045190** | 1 | 130 | 15345 | 0 | 9.83 | f | cacccgcggccgcATGgcagcatggcgtggaaagacta |
|  |  |  |  |  |  |  | r | gcgctcgagTCAtttattacaatcattccttcctccgc |
| **VIR35, putative** | **PVX_067190** | 1 | 235 | 28093 | 2 | 10.14 | f | cacccgcggccgcATGaaatatgaaaaatatggagcct |
|  |  |  |  |  |  |  | r | gcgctcgagTTAtaaaatgcttttacagagacgaca |
| **VIR8-like** | **PVX_086890** | 1 | 271 | 32043 | 0 | 9.74 | f | cacccgcggccgcATGgacagagaattaagcgaggatg |
|  |  |  |  |  |  |  | r | gcgctcgagTTAcataaaatgaagagatacatttcta |
| **VIR12, putative** | **PVX_090290** | 1 | 288 | 33994 | 1 | 6.16 | f | cacccgcggccgcATGtataaaaaattggaagatcaaaaag |
|  |  |  |  |  |  |  | r | gcgctcgagTTAttcacataaaacatcgtttttgattac |
| **VIR14-related** | **PVX_101615** | 1 | 361 | 42446 | 2 | 6.83 | f | cacccgcggccgcATGcaagaaaatgatttaaagcatttac |
|  |  |  |  |  |  |  | r | gcgctcgagCTAgacttgcactaagatagaaagggga |
| **VIR12, putative** | **PVX_106220** | 1 | 481 | 54047 | 1 | 5.17 | f | cacccgcggccgcATGtatacattatactgcttacttttgc |
|  |  |  |  |  |  |  | r | gcgctcgagTCAtaaagtatgatgtgcaattaccctg |
| **VIR18, putative** | **PVX_112125** | 1 | 423 | 45356 | 0 | 6.6 | f | cacccgcggccgcATGacgtatggacgaactagaccacg |
|  |  |  |  |  |  |  | r | gcgctcgagCTAatttaaagatccgatgagttttgaac |
| **VIR24-related** | **PVX_115485** | 1 | 302 | 36724 | 1 | 6.94 | f | cacccgcggccgcATGtgtaattttttttattccattaat |
|  |  |  |  |  |  |  | r | gcgctcgagTTAaaaactttttaccttgtaaaatagga |
| **VIR12-like** | **PVX_241290** | 1 | 771 | 89418 | 2 | 8.44 | f | cacccgcggccgcATGgagaaaaaactatcaattattaatg |
|  |  |  |  |  |  |  | r | gcgctcgagTTAtaccttataatacatgaaaagcatgg |

aa: number of aminoacids of the proteins expressed. MW: molecular weight of the protein expressed. TM: number of transmembrane domains. pI: isoelectric point of the protein. f and r: forward and reverse. The primers were designed so they included restriction sites NotI (5’) and XhoI (3’), allowing sub-cloning of PCR products in “pIVEX” vector.
