# Supplementary material for: Plasmodium vivax VIR Proteins Are Targets of Naturally-Acquired Antibody and T Cell Immune Responses to Malaria in Pregnant Women
Source: PLoS Negl Trop Dis. 2016 Oct 6;10(10):e0005009. doi: 10.1371/journal.pntd.0005009 (PMC5053494; doi:10.1371/journal.pntd.0005009)
Supplement: S2 Table — (DOCX) [file pntd.0005009.s004.docx]

**S2 Table**. **Characteristics of the *var2csa*-like *vir* genes and proteins selected for cloning and expression, and primer sequences used for gDNA amplification.**

| **name** | **id** | **exons** | **aa** | **MW** | **TM** | **pI** | **primer sequence** | **VAR2CSA homology^a^** |
| --- | --- | --- | --- | --- | --- | --- | --- | --- |
| **VIR2/15 like** | **PVX_107750** | 3 | 301 | 35945 | 1 | 9.1 | f: cacccgcggccgcATGtacaaatatgtgaaatgcttcc  r: gcgctcgagTTActtatacaaaataaataaaatg | 19% (16/85 identities) protein homology with PF3D7DBL2X |
| **VIR24-like** | **PVX_093720** | 3 | 496 | 59555 | 1 | 8.54 | f: cacccgcggccgcATGgaagtcctttcaaatgatg  r: gcgctcgagTTActtatagtaaataaaaaaaatcaacaatgctccc | 26% (21/81 identities) protein homology with PFA4DBL3X |
| **VIR5-related** | **PVX_124715** | 4 | 528 | 62862 | 0 | 8.22 | f: cacccgcggccgcATGtatgacatattcgacgatataaag  r: gcgctcgagTTAaattgaattgcatgcatcttc | 19% (16/85 identities) protein homology with PF3D7DBL2X |
| **VIR23 putative** | **PVX_069690** | 3 | 327 | 38444 | 0 | 8.59 | f: cacccgcggccgcATGaataaacttttagattc  r: gcgctcgagTTAatttattatatgttctggatc | 31% (26/85 identities) protein homology with PF3D7DBL2X |

aa: number of aminoacids of the whole VIR protein. MW: molecular weight of the whole protein. TM: number of transmembrane domains. pI: isoelectric point of the protein. f and r: forward and reverse. Primers were designed to amplify exon number 2 of the protein; ATG and TAA codons were added to start/finalize translation.

^a^ protein homology calculated as number of aminoacids in vir protein exon 2 matching with the VAR2CSA corresponding domain.
