# Supplementary material for: Plasmodium vivax VIR Proteins Are Targets of Naturally-Acquired Antibody and T Cell Immune Responses to Malaria in Pregnant Women
Source: PLoS Negl Trop Dis. 2016 Oct 6;10(10):e0005009. doi: 10.1371/journal.pntd.0005009 (PMC5053494; doi:10.1371/journal.pntd.0005009)
Supplement: S3 Table — (DOCX) [file pntd.0005009.s005.docx]

**S3 Table. Baseline characteristics of the study population***

|  | | **Site** | | | | | **p-value** |
| --- | --- | --- | --- | --- | --- | --- | --- |
|  |  | **Brazil** | **Colombia** | **Guatemala** | **India** | **PNG** |  |
| **Age (years)^a^** |  | 23.28 (5.98) [187] | 21.55 (5.66) [258] | 24.25 (7.03) [195] | 23.17 (3.18) [174] | 25.22 (5.53) [341] | < 0.0001^b^ |
| **Parity^c^** | **[0]** | 54 (29%) | 94 (36%) | 67 (34%) | 88 (51%) | 149 (43%) | < 0.0001^d^ |
|  | **[1-3]** | 91 (49%) | 118 (46%) | 69 (35%) | 80 (46%) | 124 (36%) |  |
|  | **[4+]** | 41 (22%) | 46 (18%) | 59 (30%) | 6 (3%) | 72 (21%) |  |
| **Gestational age^e^ (weeks)^a^** |  | 22.86 (8.22) [181] | 20.87 (9.39) [258] | 26.83 (7.77) [191] | 24.65 (6.54) [174] | 22.42 (4.56) [348] | < 0.0001^b^ |
| **Haemoglobin^a,e^** |  | 11.43 (1.42) [186] | 11.16 (1.66) [255] | 11.20 (1.34) [170] | 9.46 (1.67) [174] | 9.42 (1.59) [340] | < 0.0001^b^ |
| **Birth weight^f^ (g)^a^** |  | 3196 (504) [119] | 3174 (429) [136] | 3129 (631) [113] | 2888 (455 [111] | 2810 (648) [266] | < 0.0001^d^ |

*All women included in the study at the all timepoints

^a^ Arithmetic Mean (standard deviation) [number].

^b^ ANOVA.

^c^ number (percentage).

^d^ Chi-squared test.

^e^ at recruitment.

^f^ birth weight excluding twins.

PNG: Papua New Guinea.
