# Supplementary material for: Plasmodium vivax VIR Proteins Are Targets of Naturally-Acquired Antibody and T Cell Immune Responses to Malaria in Pregnant Women
Source: PLoS Negl Trop Dis. 2016 Oct 6;10(10):e0005009. doi: 10.1371/journal.pntd.0005009 (PMC5053494; doi:10.1371/journal.pntd.0005009)
Supplement: S4 Table — (DOCX) [file pntd.0005009.s006.docx]

**S4 Table. Number of samples for which anti-VIR antibody responses were analyzed by country, timepoint and antigen.**

| **Antigens** | **Timepoint** | **Brazil** | **Colombia** | **Guatemala** | **India** | **PNG** |
| --- | --- | --- | --- | --- | --- | --- |
| **VIR25 VIR5 PvLP1 PvLP2** | **R** | 133 | 217 | 173 | 134 | 137 |
|  | **D** | 75 | 117 | 105 | 98 | 134 |
|  | **P** | 38 | 10 | 60 | 5 | 55 |
| **VIR14 VIR2 VIR24** | **R** | 13 | 28 | 30 | 24 | 31 |
|  | **D** | 8 | 21 | 30 | 23 | 31 |
|  | **P** | 13 | 10 | 27 | 5 | 30 |

R: recruitment; D: delivery; P: postpartum. PNG: Papua New Guinea
