# Supplementary material for: Plasmodium vivax VIR Proteins Are Targets of Naturally-Acquired Antibody and T Cell Immune Responses to Malaria in Pregnant Women
Source: PLoS Negl Trop Dis. 2016 Oct 6;10(10):e0005009. doi: 10.1371/journal.pntd.0005009 (PMC5053494; doi:10.1371/journal.pntd.0005009)
Supplement: S5 Table — (DOCX) [file pntd.0005009.s007.docx]

**S5 Table. Correlation of anti-VIR responses with other anti-*Plasmodium* responses.**

|  | **VIR24** | **VIR25** | **VIR5** | **VIR2** | **VIR14** | **PvLP1** | **PvLP2** |  |  |
| --- | --- | --- | --- | --- | --- | --- | --- | --- | --- |
| **PvCSP** | **0.45** | **0.08** | **0.10** | -0.10 | **-0.22** | **0.69** | **0.43** |  | 0-\|0.19\| |
| **PvCSP-N** | -0.01 | **0.16** | **0.22** | 0.11 | 0.06 | **0.50** | **0.59** |  | \|0.2\|-\|0.39\| |
| **PvCSP-C** | 0.03 | **0.17** | **0.22** | -0.05 | -0.16 | **0.68** | **0.61** |  | \|0.4\|-\|0.59\| |
| **PvCSP-R** | -0.07 | **0.12** | **0.15** | -0.12 | **-0.19** | **0.71** | **0.51** |  | \|0.60\|-0.79 |
| **PvDBP** | 0.11 | -0.03 | 0.03 | 0.01 | 0.00 | **0.52** | **0.59** |  | \|0.80\|-\|1\| |
| **PvMSP1_19_** | 0.11 | 0.01 | **0.12** | 0.16 | **0.15** | **0.39** | **0.49** |  |  |
| **Pv200L** | **0.24** | **0.12** | **0.22** | **0.25** | **0.21** | **0.47** | **0.62** |  |  |
| **PvMSP1-N** | **0.37** | **0.25** | **0.37** | **0.39** | **0.45** | **0.15** | **0.22** |  |  |
| **PvMSP-5** | **0.27** | **0.21** | **0.31** | **0.57** | **0.55** | **0.17** | **0.23** |  |  |
| **PfMSP1_19_** | **0.24** | **0.13** | **0.24** | **0.26** | **0.24** | **0.42** | **0.58** |  |  |
| **PfAMA-1** | **0.23** | **0.13** | **0.25** | 0.17 | **0.18** | **0.38** | **0.59** |  |  |
| **PfEBA-175** | **0.25** | **0.10** | **0.21** | **0.19** | **0.21** | **0.38** | **0.58** |  |  |
| **PfDBL3x** | 0.10 | **0.15** | **0.22** | 0.01 | -0.06 | **0.68** | **0.69** |  |  |
| **PfDBL5ε** | **0.18** | **0.50** | **0.56** | **0.70** | **0.67** | 0.08 | 0.17 |  |  |
| **PfDBL6ε** | 0.15 | **0.13** | **0.22** | 0.00 | -0.05 | **0.59** | **0.68** |  |  |

Pv: *P. vivax*. Pf: *P. falciparum*. CSP: circumsporozoite protein; CSP-N (residues 20-96), CSP-C (residues 301–372) and CSP-R (3 tandem-repetitions of the residues 96–104). MSP: Merozoite surface protein; MSP1_19_ : 19 kDa C-terminal region, residues 1639-1729; 200L: amino acid residues 121–416 of PvMSP1; MSP1-N (fragment 170-675). DBP: Duffy binding protein, receptor binding domains – RII. AMA-1: N-terminal ectodomain of Apical membrane antigen 1. EBA_175_: erythrocyte binding protein, the receptor-binding domain-PfF2. DBL: duffy binding like proteins (VAR2CSA domains).
