# Supplementary material for: Plasmodium vivax VIR Proteins Are Targets of Naturally-Acquired Antibody and T Cell Immune Responses to Malaria in Pregnant Women
Source: PLoS Negl Trop Dis. 2016 Oct 6;10(10):e0005009. doi: 10.1371/journal.pntd.0005009 (PMC5053494; doi:10.1371/journal.pntd.0005009)
Supplement: S6 Table — (DOCX) [file pntd.0005009.s008.docx]

**S6 Table. Effect of time of bleeding on antibody levels.**

|  |  | **Fixed part** | | | |
| --- | --- | --- | --- | --- | --- |
|  | **Timepoint** | **PD** | **95% CI** | **p-value** | **p (Wald)** |
| **VIR14** | R | 1 | - | - | 0.267 |
|  | D | 1.35 | 0.90; 2.03 | 0.149 |  |
|  | P | 1.34 | 0.86; 2.10 | 0.198 |  |
| **VIR25** | R | 1 | - | - | 0.112 |
|  | D | 1.21 | 0.81; 1.83 | 0.351 |  |
|  | P | 1.62 | 1.03; 2.55 | **0.036** |  |
| **VIR24** | R | 1 | - | - | 0.069 |
|  | D | 1.55 | 1.07; 2.26 | **0.021** |  |
|  | P | 1.18 | 0.78; 1.78 | 0.439 |  |
| **VIR2** | R | 1 | - | - | 0.133 |
|  | D | 1.23 | 0.82; 1.84 | 0.327 |  |
|  | P | 1.59 | 1.01; 2.49 | **0.045** |  |
| **VIR5** | R | 1 | - | - | 0.221 |
|  | D | 1.32 | 0.89; 1.95 | 0.164 |  |
|  | P | 0.92 | 0.60; 1.42 | 0.720 |  |
| **PvLP1** | R | 1 | - | - | **0.050** |
|  | D | 1.01 | 0.87; 1.16 | 0.937 |  |
|  | P | 1.2 | 1.02; 1.40 | **0.024** |  |
| **PvLP2** | R | 1 | - | - | 0.596 |
|  | D | 0.97 | 0.85; 1.11 | 0.658 |  |
|  | P | 1.05 | 0.91; 1.22 | 0.520 |  |

Mixed-effect multi level regressions were estimated (PD: proportional difference). Timepoint (R: recruitment, D: Delivery, P: postpartum) was estimated as the fixed independent variable, and inter-site (country of origin) and inter-subject variability were estimated as random parts (not shown).
