# Supplementary material for: Plasmodium vivax VIR Proteins Are Targets of Naturally-Acquired Antibody and T Cell Immune Responses to Malaria in Pregnant Women
Source: PLoS Negl Trop Dis. 2016 Oct 6;10(10):e0005009. doi: 10.1371/journal.pntd.0005009 (PMC5053494; doi:10.1371/journal.pntd.0005009)
Supplement: S7 Table — P. vivax long synthetic peptides (PvLP) representing conserved central core (PvLP1) and C-terminal (PvLP2) VIR motifs. Derivatized diethylene glycol (DEG,Merck Chemicals,Nottingham, UK) was inserted in between the different individual segments. (DOCX) [file pntd.0005009.s009.docx]

**S7 Table**: **Peptide sequences of the long peptides (PvLP).**

| **PvLP1**  VKELCKKLVRNLKKIS—DEG—CIYLNYWLYDQ—DEG—KERKDLHDY FKNYDTIKC—DEG—CEKYCTYVTYIKSLYE—DEG—YDPKDLLSKLDC |
| --- |
| **PvLP2**  IADSPGTLGTVHEELDSNFFRNIIM—DEG—VVGVMMTFFFLYKFT—DEG—VGAFFRGGRGRVHRIPRSFHGQFPG—DEG—KRKGKIFEHNYYEEYEKEL AMYGSE—DEG—FLDSQMDRYYLNYQPDQDSYY |

*P. vivax* long synthetic peptides (PvLP) representing conserved central core (PvLP1) and C-terminal (PvLP2) VIR motifs. Derivatized diethylene glycol (DEG, Merck Chemicals, Nottingham, UK) was inserted in between the different individual segments.
